# Antegrade slow pathway mapping of typical atrioventricular nodal reentrant tachycardia based on direct slow pathway capture

**DOI:** 10.1002/joa3.12484

**Published:** 2020-12-24

**Authors:** Takeshi Tobiume, Ritsushi Kato, Tomomi Matsuura, Kazuhisa Matsumoto, Motoki Hara, Nobuyuki Takamori, Yoshio Taketani, Keisuke Okawa, Takayuki Ise, Kenya Kusunose, Koji Yamaguchi, Shusuke Yagi, Daijyu Fukuda, Hirotsugu Yamada, Tetsuzo Wakatsuki, Takeshi Soeki, Masataka Sata, Kazuo Matsumoto

**Affiliations:** ^1^ Department of Cardiology Tokushima University Hospital Tokushima Japan; ^2^ Department of Cardiology Saitama Medical University International Medical Center Hidaka Japan; ^3^ Department of Cardiology Kawashima Hospital Tokushima Japan; ^4^ Department of Cardiology Shikoku Medical Center for Children and Adults Zentsuji Japan; ^5^ Hara Clinic Higashi‐Hiroshima Higashi‐Hiroshima Japan; ^6^ Department of Cardiology Kagawa Prefectural Central Hospital Takamatsu Japan; ^7^ Department of Internal Medicine Higashi‐Matsuyama Medical Association Hospital Higashimatsuyama Japan

**Keywords:** antegrade slow pathway, atrioventricular nodal reentrant tachycardia, extrastimulation, reset

## Abstract

**Background:**

Radiofrequency (RF) ablation of typical atrioventricular nodal reentrant tachycardia (tAVNRT) is performed without revealing out the location of antegrade slow pathway (ASp). In this study, we studied a new electrophysiological method of identifying the site of ASp.

**Methods:**

This study included 19 patients. Repeated series of very high‐output single extrastimulations (VhoSESts) were delivered at the anatomical slow pathway region during tAVNRT. Tachycardia cycle length (TCL), coupling interval (CI), and return cycle (RC) were measured and the prematurity of VhoSESts [ΔPM (= TCL – CI)] and the prolongation of RCs [ΔPL (= RC – TCL)] were calculated. Pacing sites were classified into two categories: (i) ASp capture sites [DSPC(+) sites], where two different RCs were shown, and ASp non‐capture sites [DSPC(‐) sites], where only one RC was shown. RF ablation was performed at DSPC(+) sites and/or sites with catheter‐induced mechanical trauma (CIMT) to ASp.

**Results:**

DSPC(+) sites were shown in 13 patients (68%). RF ablation was successful in all patients without any degree of atrioventricular block nor recurrence. Total number of RF applications was 1.8 ± 1.1. Minimal distance between successful ablation sites and DSPC(+)/CIMT sites and His bundle (HB) electrogram recording sites was 1.9 ± 0.8 mm and 19.8 ± 6.1 mm, respectively. ΔPL of more than 92.5 ms, ΔPL/TCL of more than 0.286, and ΔPL/ΔPM of more than 1.565 could identify ASp with sensitivity of 100%, 91.1%, and 88.9% and specificity of 92.9%, 97.0%, and 97.6%, respectively.

**Conclusions:**

Sites with ASp capture and CIMT were close to successful ablation sites and could be useful indicators of tAVNRT ablation.

## Graphical abstract

A case of Direct Slow Pathway Capture (DSPC) during typical atrioventricular nodal reentrant tachycardia.

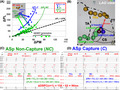

## 1 INTRODUCTION

ParaHisian pacing reveals the type of ventriculoatrial conducting pathway using the difference of pacing threshold between His bundle (HB) plus right ventricle (RV) [high pacing threshold (HPT)] and only RV [low pacing threshold (LPT)].^1^ Previous studies of typical slow‐fast form atrioventricular nodal reentrant tachycardia (tAVNRT) revealed that antegrade slow pathway (ASp) had higher pacing threshold than right atrium (RA) and could be captured only by very high‐output single extrastimulation (VhoSESt) delivered in the very vicinity of the ASp.^2^, ^3^ This study, like ParaHisian pacing, aimed to reveal the site of ASp using the difference of pacing threshold between ASp (HPT) and RA (LPT) by repeated series of VhoSESts delivered in the vicinity of the ASp during tAVNRT.

## 2 METHODS

### 2.1 Hypothesized model of tAVNRT

**FIGURE 1:**
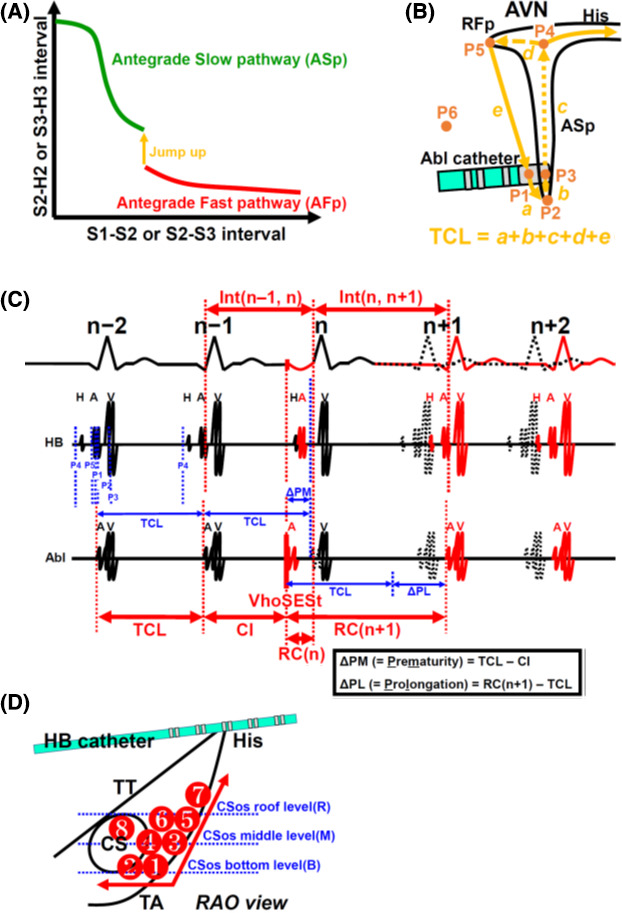
Schematic presentation of the hypothesized model of tAVNRT (A, B), effect of a VhoSESt during tAVNRT (C), and the order of manipulating Abl catheter (D). A: ASp conduction curve is hypothesized to be a sigmoid conduction curve. B: P1, P2, P3, P4, and P5 were located on the circuit of tAVNRT and P6 was located outside of the tAVNRT circuit. TCL was defined as the summation of the conduction time of *a*, *b*, *c*, *d*, and *e*. C: TCL, CI, and RC(n + 1) were measured and ΔPM and ΔPL were calculated. The electrograms drawn by the black broken line show the expected electrogram location in a case without VhoSESt. D: Abl catheter was moved sequentially from ① to ⑧ until a successful ablation was achieved. Asp, antegrade slow pathway; AFp, antegrade fast pathway; RFp, retrograde fast pathway; AVN, atrioventricular node; Abl, ablation; VhoSESt, very high‐output single extrastimulation; P1, site of RA capture by a VhoSESt delivered from Abl catheter; P2, junctional site between RA and ASp; P3, site of ASp capture by a VhoSESt delivered from Abl catheter; P4, turnaround site between ASp and RFp in AVN; P5, junctional site between RFp and RA; P6, any point in atrium and coronary sinus (CS) located away from ASp; HB, His bundle; TCL, tachycardia cycle length; CI, coupling interval, RC, return cycle; RC(n) = interval between VhoSESt and onset of QRS wave; RC(n + 1) = interval between VhoSESt and onset of A wave recorded at Abl catheter; Int(n − 1, n) = interval between the onset of QRS wave of n − 1 and n beats; Int(n, n + 1) = interval between the onset of QRS wave of n and n + 1 beats; ΔPM, prematurity of VhoSESts [= TCL − CI]; ΔPL, prolongation of return cycle [= RC(n + 1) − TCL]; TCL, tachycardia cycle length; TT, tendon of Todaro; TA, tricuspid annulus; CSos, CS ostium

**FIGURE 2:**
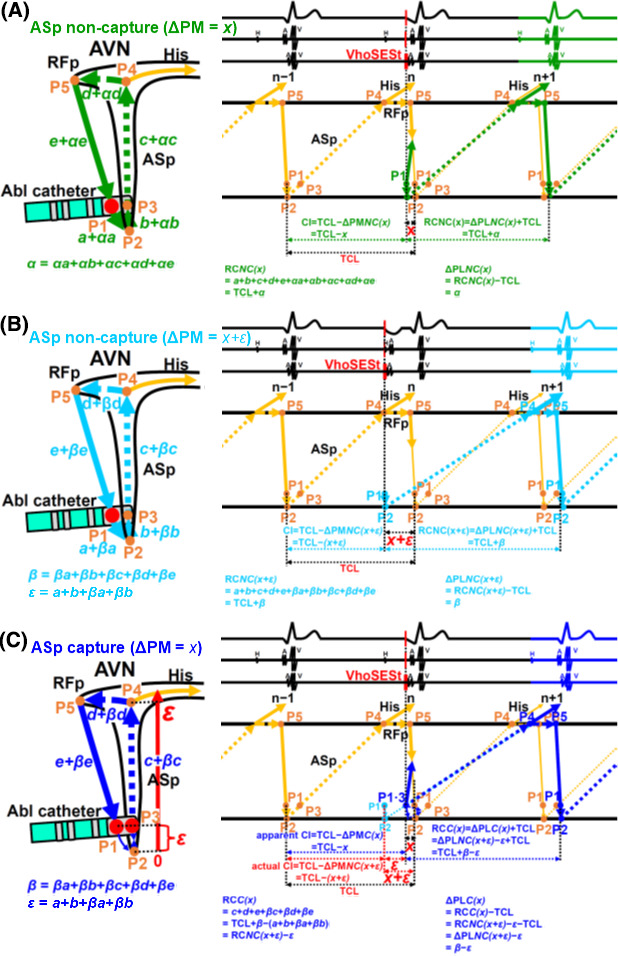
Schematic presentation of RC(n + 1) in the cases of ASp non‐capture with a ΔPM of *x* (A), of ASp non‐capture with a ΔPM of *x* + *ε* (B), and of ASp capture with a ΔPM of *x* (C). A: RC*NC(x)* = TCL + *α* and ΔPL*NC(x)* = *α*. B: RC*NC(x + ε)* = TCL + *β* and ΔPL*NC(x + ε)* = *β*. C: RC*C(x)* = TCL + *β* – *ε* = RC*NC(x + ε)* – *ε* and ΔPL*NC(x)* = *β* – *ε* = ΔPL*NC(x + ε)* – *ε*. RC*NC(x)* and RC*NC(x + ε)* = RC in the cases of ASp non‐capture with a ΔPM of *x* and *x* + *ε*, respectively; ΔPL*NC(x)* and ΔPL*NC(x + ε)* = ΔPL in the cases of ASp non‐capture with a ΔPM of *x* and *x* + *ε*, respectively; RC*C(x)* and ΔPL*C(x)* = RC and ΔPL in case of ASp capture with a ΔPM of *x*. The other abbreviations are the same as those given in Figure 1

At first, the following three preconditions were set:

- Conduction curve of the ASp corresponding to the extrastimulations with a gradual shortening of the coupling interval exhibits a sigmoid curve (illustrated in Figure 1A and patients with sigmoid curves in Figure S1).
- Conduction properties of ASp, including conduction velocity, conduction delay, and refractory period, are uniform over the entire area of the ASp (illustrated in Figure 2).
- Reentrant circuit of tAVNRT contains atrial muscle (illustrated in Figure 1B).

As shown in Figure 1B, reentrant circuit of tAVNRT was divided into five parts: P1‐P2, P2‐P3, P3‐P4, P4‐P5, and P5‐P1. Conduction time of each part was defined as *a*, *b*, *c*, *d*, and *e*, respectively. Then, tachycardia cycle length (TCL) was shown as follows:

(1) TCL=a+b+c+d+e

Here,

1. P1: site of RA capture by a VhoSESt delivered from the ablation catheter (Abl).
2. P2: junctional site between RA and ASp.
3. P3: site of ASp capture by a VhoSESt delivered from Abl catheter.
4. P4: turnaround site between ASp and retrograde fast pathway (RFp) in the atrioventricular node (AVN).
5. P5: junctional site between RFp and RA.
6. P6: any point in RA or coronary sinus (CS) located away from the ASp.

### 2.2 Effects of VhoSESts on the hypothesized model of tAVNRT

#### 2.2.1 In the case of VhoSESts delivered following the effective refractory period (ERP) of ASp(n) between n − 1 and n beats (areas ⑦, ⑧, and ⑨ in Figure 3B)

**FIGURE 3:**
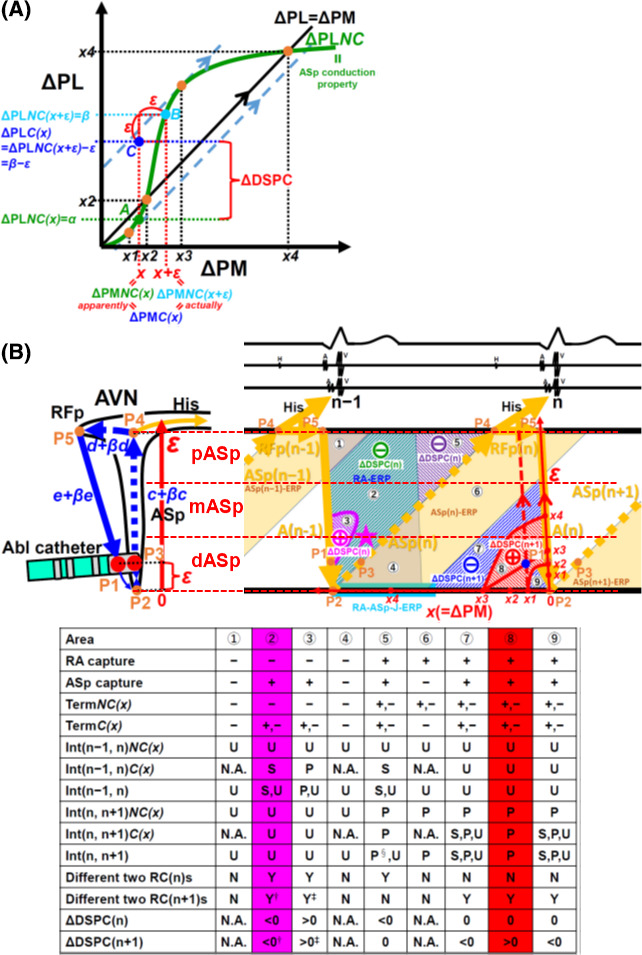
Schematic presentation of the relationship between ASp capture and ASp non‐capture in the sigmoid conduction curve of ΔPL*NC* in the ΔPM‐ΔPL coordinate system (A) and the relationship of the anatomical location of the pacing site of ASp, ΔPM, and ΔDSPC in response to a VhoSESt delivered between n − 1 and n beats (B). A: ΔPL*C(x)* (point *C*) was created by shifting ΔPL*NC(x + ε)* (point *B*) leftward then downward by the same amount of *ε*. B: The area between n − 1 and n beats was divided into nine areas based on the pacing site along the ASp, ΔPM, and ERP of RA and ASp. The effects of VhoSESts in each area were shown below. A positive value of ΔDSPC(n + 1) with a prolongation of Int(n, n + 1) was only shown in area ⑧ and was mostly confined to dASp. This area was the target of this study. The blue circle in area ⑧ showed the maximum positive value of ΔDSPC(n + 1). The pink star in area ② was the estimated pacing site in the previous study. †: the change in RC(n + 1) and ΔDSPC(n + 1) depended on the change in Int(n − 1, n). ‡: the change in RC(n + 1) and ΔDSPC(n + 1) depended on the change in Int(n − 1, n). §: the change of Int(n, n + 1) depended on the change in Int(n − 1, n). pASp, mASp, and dASp, proximal, middle, and distal part of the ASp, respectively; ERP, effective refractory period; RA‐ASp‐J, junctional site between the RA and ASp; Term, termination of tAVNRT; ΔDSPC(n) = Int(n − 1, n)*C(x)* − Int(n − 1, n)*NC(x)*; ΔDSPC(n + 1) = RC*C(x)* − RC*NC(x)* = ΔPL*C(x)* − ΔPL*NC(x)* = Int(n, n + 1)*C(x)* − Int(n, n + 1)*NC(x)* (except for area ⑤). U, unchanged; S, shortening; P, prolongation; NA = not assessed; N, no; Y, yes. The other abbreviations are the same as those given in Figures 1 and 2

Figure 1C using a ladder diagram illustrates the effects of VhoSESts on the hypothesized model of tAVNRT. During tAVNRT, a VhoSESt was delivered following the ERP of ASp(n) between n − 1 and n beats (areas ⑦, ⑧, and ⑨ in Figure 3B). TCL, coupling interval (CI), and return cycle (RC) were measured and the prematurity of VhoSESt (ΔPM) and prolongation of RC(n + 1) (ΔPL) were calculated as follows:

(2) ΔPM=TCL‐CI

(3) $$\Delta PL\;=\;RC\left(n+1\right)\;‐\;TCL$$

#### 2.2.2 Effects of VhoSESts delivered away from the ASp (Figure 1B)

If repeated series of VhoSESts were delivered at P6, VhoSESts could not capture ASp and could only capture RA, because the pacing site was away from the ASp. Therefore, RC(n + 1)s, corresponding to the same series of VhoSESts of the same CI at the same pacing site, did not change and exhibited the same single value. Such pacing sites as P6 were defined as negative sites of direct slow pathway capture [DSPC(‐) sites] (Figure 1B).

Here,

DSPC: Direct Slow Pathway Capture.

#### 2.2.3 Effects of the VhoSESts delivered in the vicinity of the ASp **(Figure 2**)

In contrast, if repeated series of VhoSESts were performed in the vicinity of the ASp, or between P2 and P4 through P3 along the ASp, these extrastimulations sometimes did not capture ASp(n + 1) (P3: HPT) and captured only RA (P1: LPT) (Figure 2A), and at the other times captured both ASp(n + 1) (P3) and RA(P1) (Figure 2C). Therefore, RC(n + 1)s [= Int(n, n + 1) + ΔPM], corresponding to the same series of VhoSESts with the same CI at the same pacing site, changed between ASp(n + 1) non‐capture [only RA (P1) capture] and both ASp(n + 1) (P3: HPT) and RA (P1: LPT) capture, and exhibited two discrete RC(n + 1)s, because conduction paths were different between those two (shown in Figure 2A,B,C, and described in detail as follows). Such pacing sites like P1 + P3 were defined as positive sites of DSPC [DSPC(+) sites].

Here,

Int(n, n + 1): interval of QRS waves between n and n + 1 beats.

#### 2.2.4 Effects of VhoSESts delivered along the ASp

As shown in Figure 2A, if repeated series of VhoSESts, delivered along the ASp with a ΔPM of *x*, did not capture ASp(n + 1) (P3: HPT) and captured only the RA (P1: LPT), RC(n + 1) [RC*NC(x)*] and ΔPL [(ΔPL*NC(x)*] were shown as follows:

(4) RCNC(x)=TCL+α

(5) $$\Delta PLNC\left(x\right)=\alpha$$

Here,

*α* (= *αa* + *αb* + *αc* + *αd* + *αe*) was the conduction delay made by a VhoSESt with a ΔPM of *x*.

*αa*, *αb*, *αc*, *αd*, and *αe* were the conduction delays made by a VhoSESt with a ΔPM of *x* at P1‐P2, P2‐P3, P3‐P4, P4‐P5, and P5‐P1, respectively.

*NC(x)*: *N*on‐*C*apture of the ASp with a ΔPM of *x*.

As shown in Figure 2B, if repeated series of VhoSESts, delivered along the ASp with a ΔPM of *x + ε,* did not capture ASp(n + 1) (P3: HPT) and captured only RA (P1: LPT), RC(n + 1) [RC*NC(x + ε)*] and ΔPL [ΔPL*NC(x + ε)*] were shown as follows:

(6) RCNC(x+ε)=TCL+β

(7) $$\Delta PLNC\left(x+\varepsilon \right)=\beta$$

Here,

*ε* (= *a* + *b* + *βa* + *βb*) was the conduction time between P1 and P3 through P2 with a ΔPM of *x + ε*.

*β* (= *βa* + *βb* + *βc* + *βd* + *βe*) was the conduction delay made by a VhoSESt with a ΔPM of *x + ε*.

*βa*, *βb*, *βc*, *βd*, and *βe* were the conduction delays made by a VhoSESt with a ΔPM of *x* + *ε* at P1‐P2, P2‐P3, P3‐P4, P4‐P5, and P5‐P1, respectively.

As shown in Figure 2C, if repeated series of VhoSESts, delivered along the ASp with a ΔPM of *x*, captured both ASp(n + 1) (P3: HPT) and RA (P1: LPT), RC(n + 1) [RC*C(x)*] and ΔPL [ΔPL*C(x)*] were shown as follows and were different from RC*NC(x)* and ΔPL*NC(x)*, respectively:

(8) RCC(x)=TCL+β‐ε=RCNC(x+ε)‐ε

(9) $$\Delta PLC\left(x\right)=\beta ‐\varepsilon =\;\Delta PLNC\left(x+\varepsilon \right)‐\varepsilon$$

Here,

*C(x)*: *C*apture of the ASp with a ΔPM of *x*.

As shown in Figure 2A,C, Abl catheter was located at the same place. Depending on the difference in the pacing threshold of ASp (P3: HPT) and RA (P1: LPT), two ΔPLs, namely ΔPL*NC(x)* and ΔPL*C(x)*, were made in the cases of ASp non‐capture (only P1 capture) and of the ASp capture (both P1 and P3 capture), respectively, in a same series of VhoSESts delivered along the ASp with a ΔPM of *x*. The difference between ΔPL*NC(x)* and ΔPL*C(x)* was defined as ΔDSPC(n + 1) and was shown as follows:

(10) $$\begin{array}{cc} \Delta DSPC\left(n+1\right) & =\Delta PLC\left(x\right)‐\;\Delta PLNC\left(x\right)\\ & =\beta ‐\varepsilon ‐\alpha =\;\Delta PLNC(x+\varepsilon )‐\;\Delta PLNC(x)‐\varepsilon \end{array}$$

As shown in Figure 3A , on the sigmoid conduction curve of ΔPL*NC* on the ΔPM‐ΔPL plane, points *A*, *B*, and *C* showed ΔPL*NC(x)*, ΔPL*NC(x + ε)*, and ΔPL*C(x)*, respectively. ΔPL*C(x)* (point *C*) was made by moving ΔPL*NC(x + ε)* (point *B*) leftward then downward by the same amount of *ε*, or moving ΔPL*NC(x + ε)* (point *B*) parallel to the straight line of ΔPL = ΔPM as a value of 2^1/2^ *ε*. The amount of *ε* became larger when Abl catheter was moved gradually from P2 (distal part of the ASp; dASp) to P4 (proximal part of the ASp; pASp) (shown in Figures 2C and 3B). Therefore, the amount of *ε* indicated the location of Abl catheter along the ASp. When the condition of *x* = *x1* and *ε* = *x3* ‐ *x1* was met, the value of ΔDSPC(n + 1) became the maximum (shown as a blue circle at area ⑧ in Figure 3B).

Here,

*x1*: the smaller value of ΔPM at the point where the slope of the sigmoid conduction curve of ΔPL*NC* was 1.

*x2*: the smaller value of ΔPM at the point where the intersection between the sigmoid conduction curve of ΔPL*NC* and the straight line of ΔPL = ΔPM occurred.

*x3*: the larger value of ΔPM at the point where the slope of the sigmoid conduction curve of ΔPL*NC* was 1.

*x4*: the larger value of ΔPM at the point where the intersection between the sigmoid conduction curve of ΔPL*NC* and the straight line of ΔPL = ΔPM occurred.

#### 2.2.5 Relationship between the value of ΔDSPC(n + 1) and pacing site

As illustrated in the ladder diagram 3B, the value of ΔDSPC(n + 1) was positive in area ⑧ and negative in areas ⑦ and ⑨. When the pacing site showed a visually identifiable large positive value of ΔDSPC(n + 1), the pacing site was suggested to be located mostly at dASp around the blue circle at area ⑧ in Figure 3B.

#### 2.2.6 In the case of VhoSESts delivered ahead of the ERP of ASp between n − 1 and n beats (areas ②, ③, and ⑤ in Figure 3B)

As shown in Figure 3B, if repeated series of VhoSESts were delivered ahead of the ERP of ASp(n) between n − 1 and n beats (areas ②, ③, and ⑤ in Figure 3B), capture or non‐capture of ASp(n) occurred and Int(n − 1, n) and RC(n) were changed. When ASp(n) was captured at areas ② and ③, where RA could not be captured because of the ERP of RA, Int(n − 1, n) and RC(n) were shortened and extended, respectively. Then, ΔDSPC(n + 1) and RC(n + 1) were shortened and extended as much as the shortening and extension of Int(n − 1, n), or RC(n), respectively, because Int(n, n + 1) was equal to TCL and was unchanged in both cases. At area ⑤, capture of ASp(n) shortened Int(n − 1, n) and RC(n) and therefore extended Int(n, n + 1). However, RC(n + 1), corresponding to a VhoSESt with the same CI at the same pacing site, had a single extended value, or ΔDSPC(n + 1) = 0, because RC(n + 1) of area ⑤ was created by RA capture, not by ASp(n) capture.

### 2.3 Patients

This study was a retrospective review of case series of 19 consecutive patients (6 men and 13 women, 61 ± 18 years, range 20 to 89 years) undergoing RF ablation of tAVNRT from June 2016 to September 2018. Written informed consent was obtained from all patients before the procedure. The study protocol was approved by the Research Ethics Committee of Tokushima University Hospital.

### 2.4 Electrophysiological study and catheter ablation

All patients underwent electrophysiological study in a fasting, unsedated state after discontinuation of all antiarrhythmic drugs for at least five half‐lives. The 12‐lead surface electrocardiograms and intracardiac electrograms from RA, CS, HB, RV, and mapping and ablation catheter (Abl) were recorded and digitally stored on a polygraph (CardioLab, GE Healthcare, WI, USA or EP Workmate, St. Jude Medical, MN, USA). Cardiac pacing was performed using a cardiac stimulator (SEC‐4103 or SEC‐5104, Nihon Kohden, Tokyo, Japan or EP‐4 stimulator, St. Jude Medical, MN, USA). All points, where HB potential were detected, where catheter‐induced mechanical trauma (CIMT) was detected, and where pacing and ablation were performed, were stored onto the three‐dimensional electroanatomical map created by the CARTO system (Biosense Webster, CA, USA) or EnSite system (St. Jude Medical, MN, USA). Dual AV nodal physiology was identified by an increment of > 50 ms of A2H2 or A3H3 interval in response to a decrement of 10 ms of A1A2 or A2A3 interval during programmed atrial stimulation. Induced AVNRT was classified into typical slow‐fast form according to the standard criteria described previously.^4^, ^5^ Radiofrequency (RF) ablation was performed using RF energy generator (Stockert; Biosense Webster, CA, USA or Ampere; St. Jude Medical, MN, USA). Endpoint for a successful ablation was Sn, S0, or S1 without the induction of tAVNRT, even during an isoproterenol infusion at a rate of 1 μg/min. The distances between the effective/ineffective ablation sites and ASp capture/CIMT sites were measured and analyzed.

Here,

Results of the RF ablation were classified as follows:

1. Sn: successful RF ablation with the elimination of ASp (without jump up)
2. S0: successful RF ablation with the inability to induce tAVNRT with jump up and no echo beat
3. S1: successful RF ablation with the inability to induce tAVNRT with jump up and one echo beat
4. F2: failure of RF ablation with the ability to induce tAVNRT with jump up and two echo beats
5. Ft: failure of RF ablation with the ability to induce tAVNRT with jump up and three or more echo beats

### 2.5 Study protocol of finding DSPC(+) site and ablation

- tAVNRT was induced by programmed stimulation. If tachycardia was terminated during mapping, tachycardia was re‐induced repeatedly.
- During tachycardia, Abl catheter was moved to the anatomical slow pathway region in the triangle of Koch and positioned at the bottom level of CS ostium (CSos). Thereafter, the order of the movement of Abl catheter is shown in Figure 1D.
- Pacing output of VhoSESt from Abl catheter was set at 10‐20 V/1 ms or at 20 mA/1 ms
- Delivery of VhoSESts was set at a timing that was earlier than the timing of the HB electrogram on HB catheter, but the precedence of which was less than 40 ms in order to exclude the possibility to retrograde intrusion of the excitation wave into the AVN when VhoSESts captured RV.
- The number of VhoSESts at each pacing site was set at 10‐15 times.
- If VhoSESts did not exhibit two visually discrete RCs with ΔDSPC(n + 1) of more than 50 ms, then Abl catheter was moved to the next pacing site in accordance with the order shown in Figure 1D.
- If VhoSESts exhibited two visually discrete RCs with ΔDSPC(n + 1) of more than 50 ms, then RF ablation was performed at that site.
- If CIMT1 occurred, the sites of CIMT1 were stored onto 3D map with reference to the fluoroscopy and CARTOREPLY in the case of using the CARTO system. Then, repeated VhoSESts to seek the sites of ASp capture were performed around these sites. If VhoSESts exhibited two visually discrete RCs, then RF ablation was performed around these sites, or if CIMT2‐4 occurred during the manipulation of Abl catheter and tAVNRT was no longer induced or sustained, then two to three RF applications were performed at and around the CIMT sites.
- If Sn, S0, or S1 was achieved even during isoproterenol infusion at a rate of 1 μg/min, RF ablation was considered to be successful. If F2 or Ft still remained, VhoSESts were continued to seek the sites of ASp capture or CIMT2‐4, by changing the pacing site in accordance with the order shown in Figure 1D until successful RF ablation was achieved.

Here,

CIMT was classified by the degree of ASp conduction disturbance as follows:

1. CIMT1: tachycardia was induced and sustained with prolongation of TCL.
2. CIMT2: tachycardia was induced but the sustenance of the tachycardia was reduced.
3. CIMT3: tachycardia was not reproducibly induced.
4. CIMT4: tachycardia was not induced with a loss of ASp conduction (loss of jump up)

### 2.6 Statistics

Continuous variables were expressed as mean ± SD, and those variables were compared using the Student's t‐test. Sensitivity, specificity, and positive and negative predictive values were calculated using standard methods. Optimal cut‐off values of the continuous variables to identify the ASp were determined using receiver operating characteristic (ROC) curve analysis. The performance of the pacing studies was evaluated according to the areas under the ROC curves. A p‐value < 0.05 was considered statistically significant.

## 3 RESULTS

### 3.1 Patient Characteristics

All 19 patients were diagnosed as tAVNRT. TCL, A‐H interval, and H‐A interval during tachycardia were 355 ± 63 ms, 320 ± 63 ms, and 35 ± 9 ms, respectively (Table 1).

**TABLE 1:** Clinical and procedural characteristics

Pt.	Age (yrs)	Sex	TCL (ms)	A–H (ms)	H–A (ms)	PO (/1 ms)	Type of CIMT	%ASp*C* (ASp*C*./total)	No. of RF	JR	Result	His–Sc. (mm)	CIMT–Sc. or ASp*C*–Sc. (mm)	CIMT–F. or ASp*C*–F. (mm)	f/u (M)	Rec.
1	69	F	322	278	44	20 V	3	26.7 (4/15)	1	(+)	S0	22.0	1.4	NA	47	(‐)
2	69	M	413	378	35	20 V	2	NA	3	(+)	S1	28.2	1.2	NA	46	(‐)
3	68	M	313	300	13	20 V	1	24.4 (11/45)	2	(+)	Sn	16.2	0.7	2.6	45	(‐)
4	44	F	262	224	38	20 V	(‐)	7.7 (1/13)	2	(+)	Sn	17.6	3.2	5.8	44	(‐)
5	83	F	442	414	28	20 V	(‐)	18.2 (4/22)	1	(+)	Sn	25.8	2.3	NA	38	(‐)
6	59	M	425	399	26	20 V	(‐)	20.8 (10/48)	1	(‐)	S1	32.7	2.7	NA	21	(‐)
7	64	F	346	324	22	20 mA	1	23.1 (3/13)	1	(+)	S1	24.0	2.6	NA	34	(‐)
8	89	F	374	335	39	20 V	3	NA	2	(+)	Sn	17.8	2.9	NA	34	(‐)
9	50	F	272	225	47	20 mA	1	44.0 (11/25)	5	(+)	Sn	13.9	1.4	2.5, 14.3, 18.6, 22.1	33	(‐)
10	31	F	258	221	37	20 V	3	NA	2	(+)	S1	19.9	1.2	NA	31	(‐)
11	76	M	422	379	43	20 mA	(‐)	23.7 (64/270)	1	(+)	Sn	15.0	1.7	NA	30	(‐)
12	59	M	296	262	34	20 mA	1	36.4 (4/11)	1	(+)	Sn	10.7	2.0	NA	29	(‐)
13	57	F	373	343	30	20 V	2	NA	2	(+)	Sn	19.3	2.5	NA	28	(‐)
14	63	F	336	303	33	20 V	(‐)	8.3 (1/12)	1	(+)	Sn	20.3	1.7	NA	27	(‐)
15	20	F	370	324	46	20 V	1	12.9 (4/31)	1	(+)	Sn	29.9	2.0	NA	26	(‐)
16	82	F	480	430	50	10 V	(‐)	16.7 (2/12)	1	(+)	Sn	16.0	2.0	NA	26	(‐)
17	48	M	358	328	30	20 mA	1	86.9 (73/84)	4	(+)	Sn	19.9	1.8	1.6, 2.7, 2.9	25	(‐)
18	75	F	314	281	33	20 V	3	NA	2	(+)	S0	14.2	2.8	NA	24	(‐)
19	53	F	364	331	33	20 V	4	NA	2	(+)	Sn	12.2	0.5	NA	20	(‐)
Mean ± SD	61 ± 18		355 ± 63	320 ± 63	35 ± 9				1.8 ± 1.1			19.8 ± 6.1	1.9 ± 0.8	8.1 ± 8.0	33 ± 8	

Abbreviations: Pt., patient; TCL, tachycardia cycle length; A‐H, Atrio‐His interval; H‐A, His‐atrial interval; PO, pacing output; CIMT, catheter‐induced mechanical trauma; Asp, antegrade slow pathway; ASp*C*, ASp capture; %ASp*C*, percentage of ASp*C*; Sc., Success; No., number; RF¸radiofrequency application; JR, junctional rhythm; Sn, successful RF with elimination of ASp; S0, successful RF with inability to induce AVNRT with no echo beat; S1, successful RF with inability to induce AVNRT with one echo beat; His − Sc., minimal distance between His bundle electrogram recording sites and successful RF sites on 3D map; CIMT − Sc., minimal distance between CIMT sites and successful RF sites on 3D map; ASp*C* − Sc., minimal distance between ASp*C* sites and successful RF sites on 3D map; F. = failure; CIMT − F., minimal distance between CIMT sites and failed RF sites on 3D map; ASp*C* − F., minimal distance between ASp*C* sites and failed RF sites on 3D map; NA, not applicable; f/u, follow‐up periods; Rec., recurrence.

### 3.2 Pacing study for the identification of the ASp

All of the settings of the pacing output of 10‐20 V/1 ms and 20 mA/1 ms showed ASp capture. All of the sites of ASp capture except for one were associated with RA capture. ASp capture was detected in 13 patients (68%). Any type of CIMT was detected in 13 (68%) and CIMT2‐4 was detected in seven (37%). Both ASp capture and CIMT2‐4 were detected in only one patient (5%). In 13 patients who showed ASp capture, 1309 VhoSESts from 105 pacing sites were performed. Among those 105 pacing sites, ASp capture was shown in 33 pacing sites (2.5 ± 3.6 pacing sites per patient) and ASp non‐capture in 72 pacing sites (5.5 ± 4.4 pacing sites per patient). Among 1309 VhoSESts, 601 (18.2 ± 12.9 VhoSESts per pacing site) were performed at DSPC(+) sites and 708 (9.8 ± 10.1 VhoSESts per pacing site) were performed at DSPC(‐) sites (Table 1). At DSPC(+) sites, A/V ratio was 0.47 ± 0.51 (range from 0.04 to 1.61).

### 3.3 Condition showing more than 50 ms of ΔDSPC(n + 1)

**FIGURE 4:**
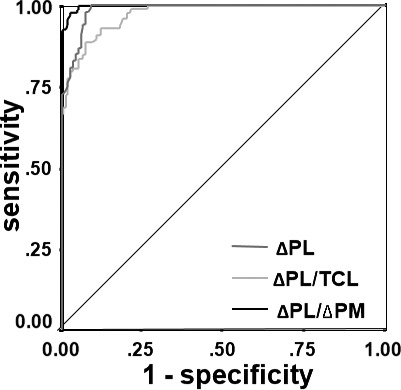
Receiver operating characteristic (ROC) curves for the detection of ASp displaying the diagnostic performance of a ΔPL > 92.5 ms, ΔPL/TCL > 0.286, and ΔPL/ΔPM > 1.565. All indicators showed a good discrimination. The other abbreviations are the same as those given in Figures 1, 2, and 3

In order to make ΔDSPC(n + 1) be identified visually, ΔDSPC(n + 1) should be more than 50 ms in response to ΔPMs with a dispersion of less than 10 ms We selected VhoSESts with ΔDSPC(n + 1)s of more than 50 ms in response to ΔPMs with a dispersion of less than 10 ms (if ΔPM of the case showing ASp capture was *x*, points of ASp non‐capture with the range of ΔPM of *x* ≤ ΔPM ≤ *x + 10* ms were chosen). This condition was satisfied in all 13 patients who showed ASp capture. Of all 601 VhoSESts, which were performed at DSPC(+) sites, 267 satisfied this condition. In those 267 VhoSESts, ASp capture was recognized in 99, and the other 168 showed ASp non‐capture. ΔPM was 63 ± 18 ms and slightly preceded the timing of HB electrogram. The value of ΔPL/ΔPM in the case of ASp non‐capture was 0.9 ± 0.2, and which was slightly less than 1. The sensitivity and specificity of ΔPL of more than 92.5 ms, ΔPL/TCL of more than 0.286, and ΔPL/ΔPM of more than 1.565 for the identification of the ASp were 100% and 91.1%, 88.9% and 92.9%, and 97.0% and 97.6%, respectively (Figure 4; Tables 2 and 3).

**TABLE 2:** Diagnostic value of ΔPL, ΔPL/TCL, and ΔPL/ΔPM for identification of ASp

		**Cut‐Off Values**	**Sensitivity (%)**	**Specificity (%)**	**PPV (%)**	**NPV (%)**	**AUC** **(95% CI)**
**tAVNRT (13 pts, 366 VhoSESts)**	**ΔPL (ms)**	**92.5**	**100**	**91.1**	**86.8**	**100**	**0.987 (0.978‐0.996)**
	**ΔPL/TCL**	**0.286**	**88.9**	**92.9**	**88.0**	**93.4**	**0.974 (0.959‐0.988)**
	**ΔPL/ΔPM**	**1.565**	**97.0**	**97.6**	**96.0**	**98.2**	**0.988 (0.968‐1.008)**

Abbreviations: Asp, antegrade slow pathway; tAVNRT, typical AVNRT; pts, patients; VhoSESts, very high‐output single extrastimulations; ΔPL, prolongation of return cycle [= RC(n + 1) − TCL]; TCL, tachycardia cycle length; ΔPM, prematurity of VhoSESts [= TCL − CI]; PPV, positive predictive value; NPV, negative predictive value; AUC, area under ROC curve; CI, confidence interval.

**TABLE 3:** Characteristics of responses against VhoSESts at the sites showing ΔDSPC(n + 1) of more than 50 ms in response to ΔPMs with dispersion of less than 10 ms

	Total (n = 267)	ASp*C* (n = 99)	ASp*NC* (n = 168)	P
ΔPM (ms)	63 ± 19	57 ± 18	67 ± 18	*P* < .0001
ΔPM/TCL	0.19 ± 0.06	0.16 ± 0.05	0.21 ± 0.06	*P* < .0001
ΔPL (ms)	NA	169 ± 44	61 ± 24	*P* < .0001
ΔPL/TCL	NA	0.45 ± 0.11	0.18 ± 0.07	*P* < .0001
ΔPL/ΔPM	NA	3.1 ± 0.9	0.9 ± 0.2	*P* < .0001

Abbreviations: VhoSESts, very high‐output single extrastimulations; Asp, antegrade slow pathway; ASp*C*, ASp capture; ASp*NC*, ASp non‐capture; ΔPM, prematurity of VhoSESts [= TCL − CI]; ΔPL, prolongation of return cycle [= RC(n + 1) − TCL]; TCL, tachycardia cycle length; ΔDSPC(n + 1) = difference of ΔPL between the cases of ASp capture and those of ASp non‐capture (=ΔPL*C* − ΔPL*NC*) of the n + 1 beat; NA = not applicable.

### 3.4 Catheter ablation

Catheter ablation was performed and finished successfully in all 19 patients. In 68% (13/19) of the patients, ASp was eliminated. The number of RF applications was 1.8 ± 1.1 (1.7 ± 1.3 in patients with ASp capture and 2.0 ± 0.6 with CIMT2‐4, respectively) and only one attempt was successful in 47% (9/19). Junctional rhythm (JR) during RF ablation occurred in all but one patient in whom RF application was performed at the bottom level of CSos. The minimal distance between the successful ablation sites and DSPC(+) sites was 2.0 ± 0.6 mm and the minimal distance between the successful ablation sites and CIMT2‐4 sites was 1.8 ± 0.9 mm. CIMT1 sites were almost the same as DSPC(+) sites. The minimal distance between the ineffective ablation sites and DSPC(+) sites was 8.1 ± 8.0 mm and was significantly larger than that between the effective ablation sites and DSPC(+) sites (1.9 ± 0.8 mm) (*P* = .01). The minimal distance between the successful ablation sites and HB electrogram recording sites was 19.8 ± 6.1 mm (20.3 ± 6.4 mm in the patients with ASp capture and 18.6 ± 5.6 mm with CIMT2‐4, respectively). There were no cases with any degree of AVB or recurrence of tAVNRT during the follow‐up period of 33 ± 8 months (Table 1).

### 3.5 Case presentation

**FIGURE 5:**
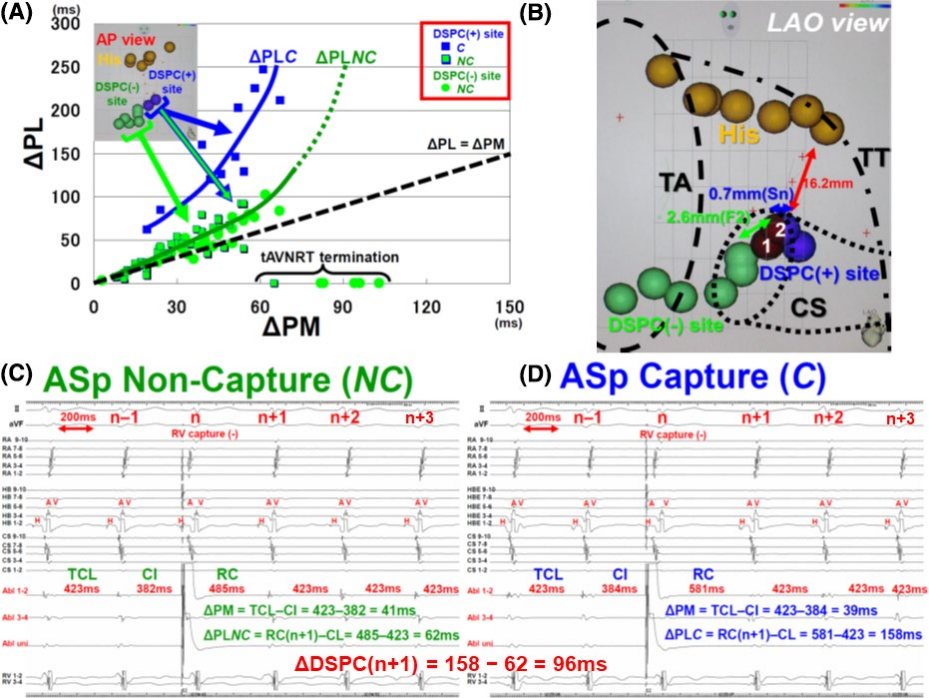
Case presentation of patient 3. A: Conduction curve of ΔPL*NC* (green) and ΔPL*C* (blue) on the ΔPM‐ΔPL coordinate system. Two discrete values of ΔPL were shown at DSPC(+) sites (ΔPL*NC* and ΔPL*C*). In the case of ASp non‐capture, tAVNRT was terminated in response to a ΔPM of more than 60 ms Blocking site was estimated to be located at RA‐ASp‐J. B: Location of DSPC(+) sites, DSPC(‐) sites, Abl sites, and HB electrogram recording sites on the 3D map. First RF application^1^ was delivered 2.6 mm blow DSPC(+) site, and JR occurred, but resulted in F2. Second RF application^2^ at DSPC(+) site was successful (Sn). C: Intracardiac electrograms in a case of ASp non‐capture. ΔPL*NC* was 62 ms in response to a ΔPM of 41 ms D: Intracardiac electrograms in a case of ASp capture at the same pacing site as C. ΔPL*C* was 158 ms in response to a ΔPM of 39 ms and ΔDSPC(n + 1) was 96 ms Notice that HB was antegradely activated by ASp(n), not by VhoSESt retrogradely. The other abbreviations are the same as those given in Figures 1, 2, and 3

In Figure 5, we present a case of ASp capture (patient 3). During the tachycardia, VhoSESts were started from the bottom level of CSos. When VhoSESts were performed at the roof level of CSos, ASp capture was detected and exhibited two different ΔPLs of 62 ms (ΔPL*NC*) and 158 ms (ΔPL*C*) (ΔDSPC of 96 ms) in response to almost the same ΔPM of 41 ms and 39 ms, respectively. TCLs of n + 1, n + 2, and n + 3 beats following VhoSESts were all unchanged to be 423 ms The first RF application performed 2.6 mm below DSPC(+) site failed in F2 with JR, and the second RF application performed at a site of 0.7 mm from DSPC(+) site was successful with JR and eliminated ASp conduction (Sn). More cases of the pacing study are shown in Figures S1, S2, S3, S4, and S6.

## 4 DISCUSSION

### 4.1 Main findings

In the present study, we showed that all of the sites with ASp capture, CIMT, and successful ablation were adjacent to each other and indicated the location of ASp. Our protocol was successful for the treatment of tAVNRT with a low number of both effective and ineffective RF applications without any degree of AVB or recurrence. A visually identifiable difference in ΔDSPC of more than 50 ms was shown in all patients with ASp capture. About one‐third of the patients had CIMT2‐4, but were successfully ablated according to a systematic manipulation of Abl catheter and 3D mapping system (especially using CARTOREPLAY).

### 4.2 Previous studies

Electrophysiological phenomenon of ASp capture has been reported in only two case reports ^2^, ^3^. In those papers, (i) resetting of tAVNRT with the shortening of Int(n, n–1) by capturing only the ASp in response to VhoSESts delivered at a timing of the ERP of both RA and RV and (ii) termination of tAVNRT according to the conduction block in the ASp in response to VhoSESts delivered at a timing of the ERP of both the RA and RV were reported. These ASp capture sites were spatially away from the HB recording sites, and catheter ablation was successful at these sites. This electrophysiological phenomena reported in these previous studies could also be explained by our model (Figure 3B), and the sites of ASp capture/successful ablation were located at the pink star in area ② in Figure 3B. Only with the electrophysiological information, previous reports could only indicate that the pacing sites were located near the ASp, but could not indicate any information about which parts of the ASp were captured, because both the termination of tAVNRT and shortening of Int(n, n–1) with the same extent could be shown not only in dASp but also in pASp and mASp in area ② of Figure 3B. And whether or not ASp capture sites were spatially away from the HB recording sites, which did not always mean dASp, was only judged by fluoroscopy or 3D map. Our method, only electrophysiologically, could identify dASp by finding out two discrete RCs with ΔDSPC of more than 50 ms Moreover, the minimal distance between the successful ablation sites and HB electrogram recording sites measured in this study (19.8 ± 6.1 mm) was longer than that in the previous study of a combined anatomical and electrogram‐guided approach (11.6 ± 4.7 mm), and this also suggested that our method could identify dASp.^6^

### 4.3 Catheter‐induced mechanical trauma (CIMT) of ASp

There were two previous studies about CIMT in patients with AVNRT, reporting that CIMT was shown in 0.4%–3.9%.^7^, ^8^ In our study, 68.4% (13/19) showed any type of CIMT during mapping of the ASp and 36.8% (7/19) could not continue pacing study (CIMT2‐4). The incidence of CIMT was obviously higher than the previous studies. This was because pacing study has been performed repeatedly and in detail at the anatomical slow pathway region in the vicinity of the ASp. However, RF ablations were successful in all seven patients of CIMT2‐4 with a low number of RF applications (2.0 ± 0.6). This was because Abl catheter has been manipulated systematically and that the sites showing CIMT have been stored onto 3D map (especially using CARTOREPLAY).

### 4.4 Sigmoid conduction property of ASp

Some previous studies actually showed a sigmoid AVN conduction curve of both the ASp^9^, ^10^, ^11^, ^12^, ^13^, ^14^, ^15^, ^16^, ^17^ and antegrade fast pathway (AFp).^20^ As shown in Figure S9, among the 13 patients showing the conduction curve of both ASp capture and ASp non‐capture, three patients (23%) exhibited the sigmoid conduction curve, and all sigmoid conduction curves were only demonstrated in the conduction curves of ASp capture. Therefore, the sigmoid conduction curves could only be seen when the extrastimulations were delivered with a quite short coupling interval and that this could be a reason why the sigmoid conduction curves could be seen only rarely. Moreover, as shown in Figure S5, if ASp conduction curve is the exponential curve, as is generally believed, the slope of ASp conduction curve is more than 1 when the value of *x* is more than *x1*. Therefore, if the value of *x* is more than *x1* and VhoSESts are delivered gradually approaching to pASp from dASp, then ΔPL*C(x)* becomes monotonically larger (Figure S5A). In patient 17 (shown in Figure S6), most proximal pacing site ④ showed the smallest ΔPL*C*, and this could not be explained by the exponential curve model, but can be explained by the sigmoid curve model (Figure S5B). However, there was the possibility that AVN conduction curve showed both the exponential and sigmoid conduction curves, and further study will be needed.

### 4.5 Validity of conditions of VhoSESt

In a previous study, pacing output of VhoSESt was reported to be 10 mA/2 ms^3^ In this study, the pacing output was set at 10‐20 V/1 ms or 20 mA/1 ms and ASp capture was shown in all conditions. Therefore, all of these conditions can be used for the detection of the ASp. In this study, the number of VhoSESts in each pacing site was set to 10‐15 times. This number of VhoSESts was thought to be appropriate, because percentage of ASp capture at DSPC(+) sites was 31.9% (192/601:26.9 ± 20.7%, range 7.7%–86.9%) and probability of at least one VhoSESt out of 10‐15 VhoSESts can capture the ASp at one DSPC(+) site was presumed to be 97.9%–99.7% (average 95.6%–99.1%, range 47.3 to 99.8% in the case of 10 VhoSESts and 61.7 to 99.9% in the case of 15 VhoSESts with two‐sided 95% confidence interval). In this study, timing of VhoSESt was set around the timing of HB electrogram. That timing was thought to be appropriate, because (i) tAVNRT did not usually terminate at that timing of VhoSESts, and (ii) ΔDSPC was mainly decided not by the apparent CI (*x*), but by the actual CI against the ASp(n + 1) (*x + ε*), and the amount of *ε*, which indicated the location of Abl catheter along the ASp, could vary tens of milliseconds in response to the movement of Abl catheter by several millimeters according to the longer delay of the ASp.

### 4.6 Application of this method

This method also could be applied to find out the ASp in patients with S/S AVNRT and we experienced two successful cases (these patients were not included in this study because their tachycardia was not typical AVNRT, data are shown in S1). However, this method cannot be applied to find out the RSp in patients with F/S AVNRT because RV can be captured at this timing and the tachycardia can be reset by the excitation wave intruding into the circuit retrogradely.

### 4.7 Limitations

Our institute is a low volume center and has a small number of patients with tAVNRT. A multicenter large volume prospective study will be needed.

Our study and the previous reports could only give explicable information about areas ② and ⑧ in Figure 3B, but gave no information about the other areas. Further study will be needed.

In this study, the conduction property of the ASp was hypothesized to be uniform over the entire area of the ASp, but this is not exactly correct, because the atrioventricular conducting system has a multilayer structure. ^19^, ^20^ However, the exact conduction property of each layer and interlayer is uncertain and controversial. Therefore, the morphology of each area in Figure 3B is likely to be changed.

In this study, all ASps were located at the anatomical slow pathway region. However, slow pathway is sometimes located at the mitral annulus, tricuspid annulus, and non‐coronary cusp of Valsalva.^21^, ^22^, ^23^, ^24^, ^25^ In such cases, whether or not our method worked well was uncertain.

In this study, none of the tAVNRTs had VA conduction block. However, the tAVNRTs sometimes had VA block according to the conduction block at the upper common pathway.^26^, ^27^, ^28^ In such cases, the tachycardia circuit contained no atrial muscle and whether or not our method worked well was uncertain.

In the anatomical slow pathway region, the autonomic nervous system has been reported to be captured sometimes and to change the conduction property of the ASp or AFp.^17^, ^29^ However, in this study, tAVNRTs were terminated only when ΔPM was large and RCs were progressively larger when ΔPM gradually became larger. Therefore, the termination and prolongation had a clear relationship with ΔPM and effects of the autonomic nervous system were uncertain.

ASp capture was shown not only during the tachycardia but also during the induction of tachycardia (data not shown). Using ASp capture during the induction of tachycardia, we may detect the ASp of patients with CIMT2‐3 and may perform basic animal study of ASp capture. Further study will be needed.

## 5 CONCLUSIONS

Sites with ASp capture and CIMT were close to successful ablation sites and could be useful indicators of tAVNRT ablation.

## CONFLICT OF INTEREST

Authors declare no conflict of interests for this article.

## DISCLOSURES

The protocol for this research project has been approved by Tokushima University Hospital's institutional review board (Approval No. 2789; Date of Approval, 27/06/2017) and it conforms to the provisions of the Declaration of Helsinki.

## Supporting information

Fig S1

Fig S2

Fig S3

Fig S4

Fig S5

Fig S6
