# Supplementary figures and images for: Antegrade slow pathway mapping of typical atrioventricular nodal reentrant tachycardia based on direct slow pathway capture
Source: J Arrhythm. 2020 Dec 24;37(1):128–39. doi: 10.1002/joa3.12484 (PMC7896471; doi:10.1002/joa3.12484)

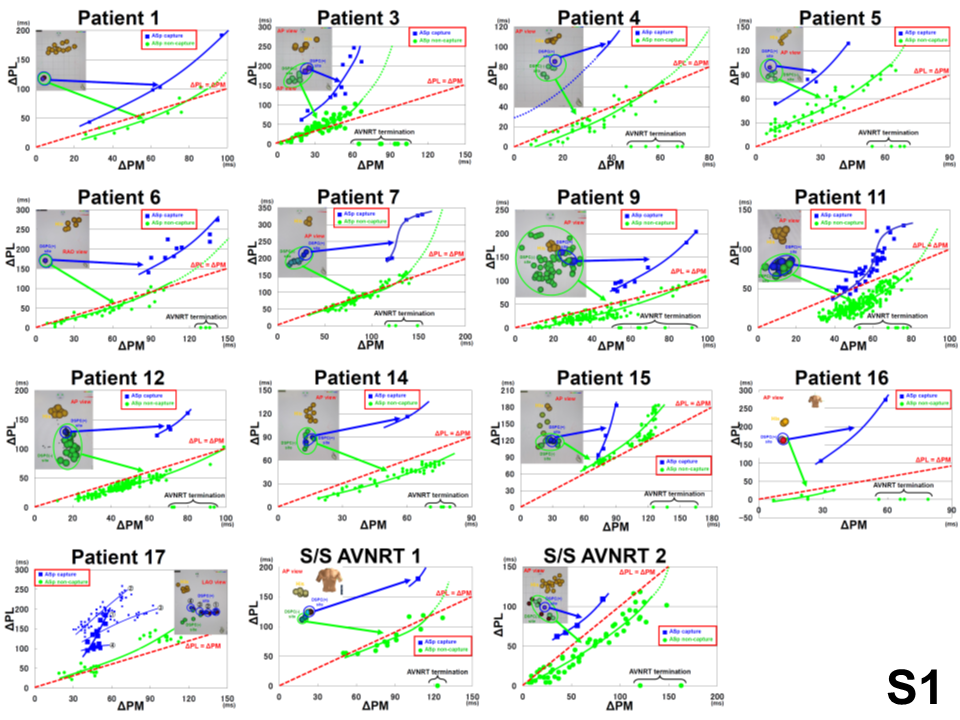

Supplement: Supplementary file 1 — Fig S1 [file JOA3-37-128-s001.TIF]

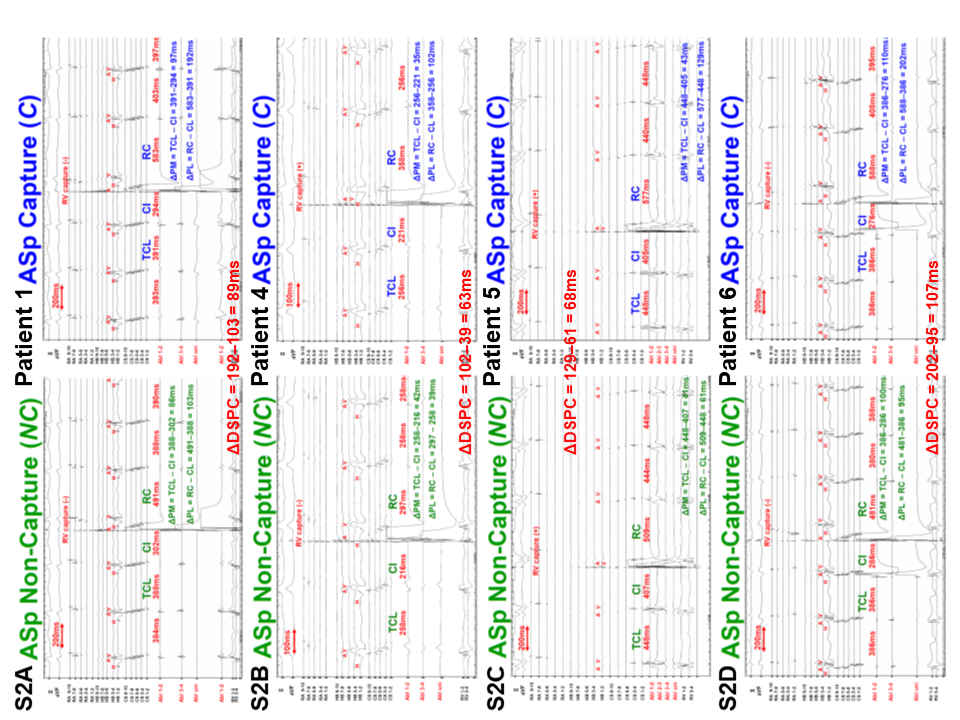

Supplement: Supplementary file 2 — Fig S2 [file JOA3-37-128-s002.TIF]

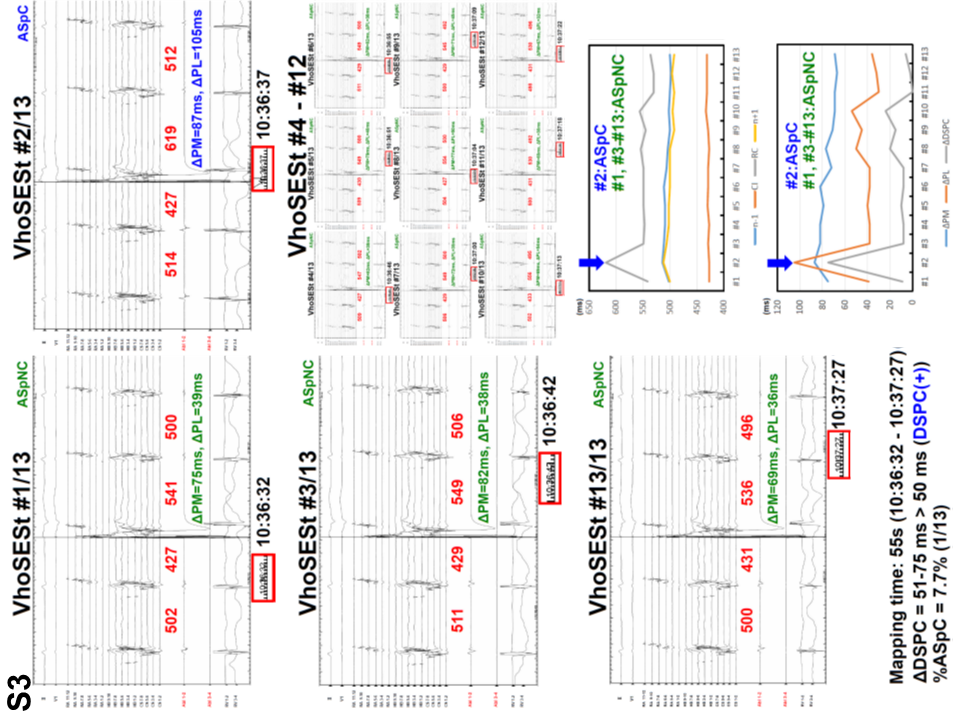

Supplement: Supplementary file 3 — Fig S3 [file JOA3-37-128-s003.TIF]

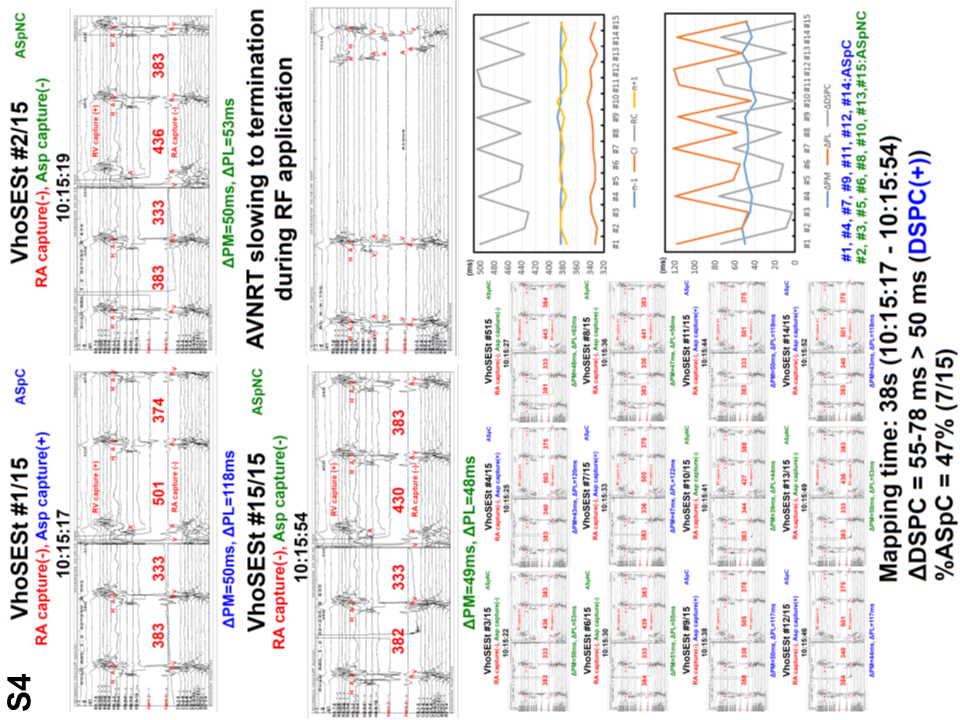

Supplement: Supplementary file 4 — Fig S4 [file JOA3-37-128-s004.TIF]

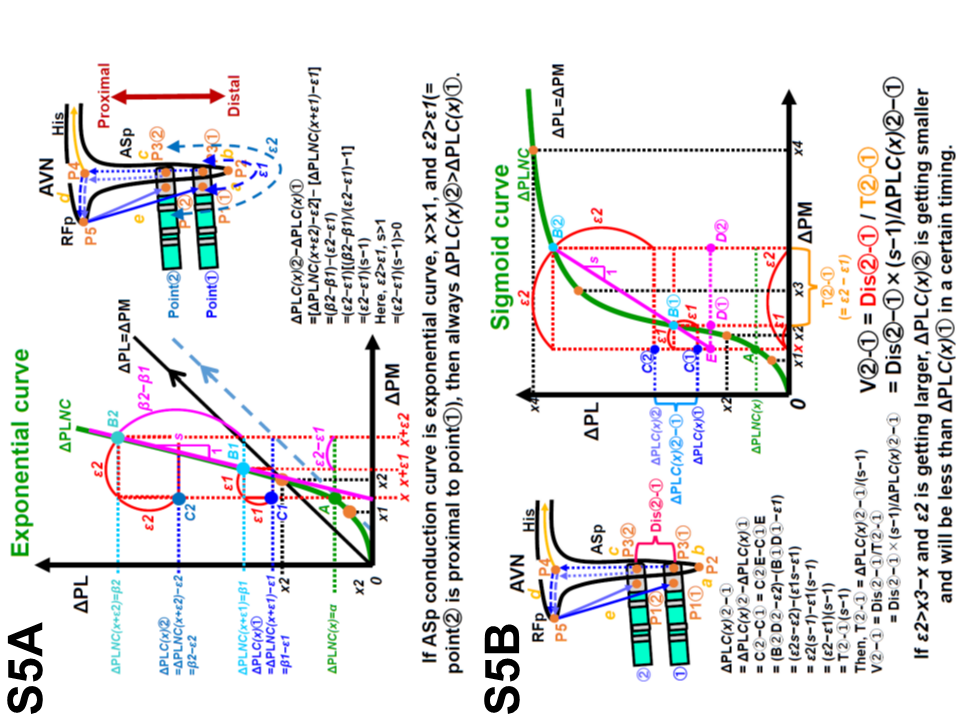

Supplement: Supplementary file 5 — Fig S5 [file JOA3-37-128-s005.TIF]

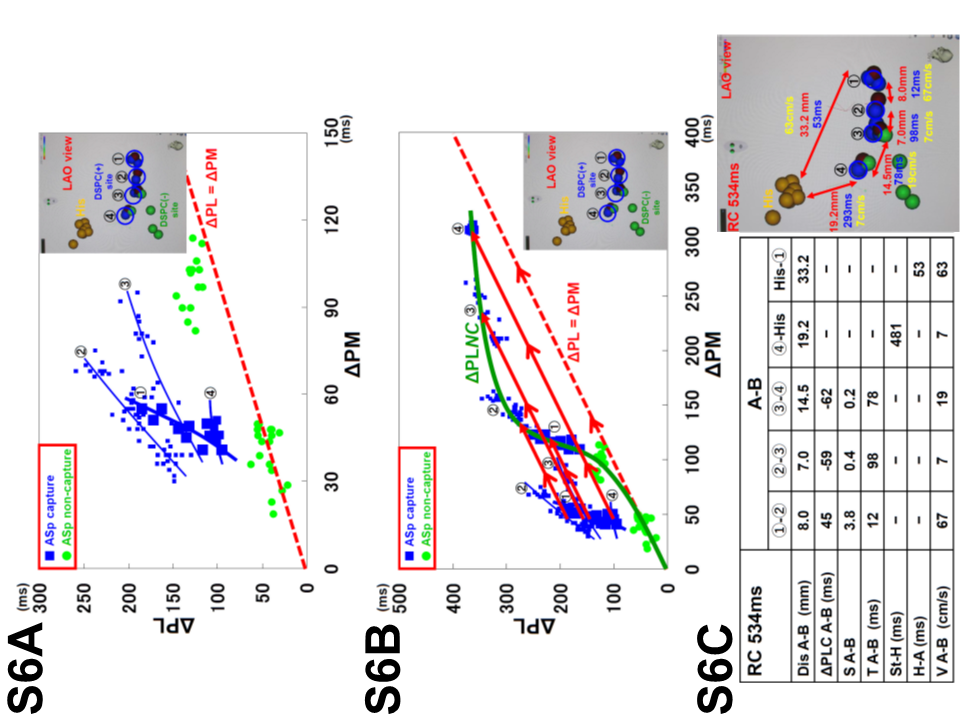

Supplement: Supplementary file 6 — Fig S6 [file JOA3-37-128-s006.tif]
